# A New Academic Quality at Work Tool (AQ@workT) to Assess the Quality of Life at Work in the Italian Academic Context

**DOI:** 10.3390/ijerph19063724

**Published:** 2022-03-21

**Authors:** Margherita Brondino, Fulvio Signore, Agnese Zambelli, Emanuela Ingusci, Silvia Pignata, Amelia Manuti, Maria Luisa Giancaspro, Alessandra Falco, Damiano Girardi, Dina Guglielmi, Marco Depolo, Barbara Loera, Daniela Converso, Sara Viotti, Andreina Bruno, Silvia Gilardi, Michela Cortini, Francesco Pace, Vincenza Capone, Silvia Platania, Margherita Zito, Margherita Pasini, Massimo Miglioretti, Giuseppina Dell’Aversana, Giuseppe Carrus, Paola Spagnoli

**Affiliations:** 1 Department of Human Sciences, University of Verona, Lungadige Porta Vittoria 17, 37129 Verona, Italy; margherita.brondino@univr.it (M.B.); margherita.pasini@univr.it (M.P.); 2 History, Society and Human Studies Department, University of Salento, Via di Valesio 24, 73100 Lecce, Italy; fulvio.signore@unisalento.it (F.S.); emanuela.ingusci@unisalento.it (E.I.); 3 Department of Education Studies “Giovanni Maria Bertin”, Alma Mater Studiorum—University of Bologna, Via Zamboni 33, 40126 Bologna, Italy; agnese.zambelli@unibo.it (A.Z.); dina.guglielmi@unibo.it (D.G.); marco.depolo@unibo.it (M.D.); 4 STEM Unit, University of South Australia, Mawson Lakes Boulevard, Mawson Lakes, SA 5095, Australia; silvia.pignata@unisa.edu.au; 5 Department of Education, Psychology, Communication, University of Bari, Palazzo Chiaia Napolitano Via Crisanzio 42, 70121 Bari, Italy; amelia.manuti@uniba.it (A.M.); maria.giancaspro@uniba.it (M.L.G.); 6 Department of Philosophy, Sociology, Education and Applied Psychology, University of Padova, Via 8 Febbraio 2, 35122 Padova, Italy; alessandra.falco@unipd.it (A.F.); damiano.girardi@unipd.it (D.G.); 7 Department of Psychology, University of Turin, Via Verdi 10, 10124 Turin, Italy; barbara.loera@unito.it (B.L.); daniela.converso@unito.it (D.C.); sara.viotti@unito.it (S.V.); 8 Department of Education Sciences, University of Genoa, 16128 Genova, Italy; andreina.bruno@unige.it; 9 Department of Labour and Welfare Studies, University of Milan, 20122 Milan, Italy; silvia.gilardi@unimi.it; 10 Department of Psychological Sciences, Health and Territory, University G. d’Annunzio of Chieti-Pescara, 66100 Chieti, Italy; michela.cortini@unich.it; 11 Department of Economic, Business and Statistic Science, University of Palermo, 90133 Palermo, Italy; francesco.pace@unipa.it; 12 Department of Humanities, University of Naples “Federico II”, 80138 Napoli, Italy; vincenza.capone@unina.it; 13 Department of Educational Sciences, Section of Psychology, University of Catania, 95124 Catania, Italy; splatani@unict.it; 14 Department of Business, Law, Economics and Consumer Behaviour “Carlo A. Ricciardi”, Università IULM, Via Carlo Bo 1, 20143 Milan, Italy; margherita.zito@iulm.it; 15 Department of Psychology, University of Milano-Bicocca, 20126 Milano, Italy; massimo.miglioretti@unimib.it (M.M.); giuseppina.dellaversana@unimib.it (G.D.); 16 Department of Cultural and Educational Studies, University of Roma Tre, Via del Castro Pretorio 20, 00185 Roma, Italy; giuseppe.carrus@uniroma3.it; 17 Psychology Department, University of Campania Luigi Vanvitelli, 81100 Caserta, Italy

**Keywords:** job demands-resources model, quality of life in academia, validation, academic teaching staff, assessment tool

## Abstract

The present study provides evidence for a valid and reliable tool, the Academic Quality at Work Tool (AQ@workT), to investigate the quality of life at work in academics within the Italian university sector. The AQ@workT was developed by the QoL@Work research team, namely a group of expert academics in the field of work and organizational psychology affiliated with the Italian Association of Psychologists. The tool is grounded in the job demands-resources model and its psychometric properties were assessed in three studies comprising a wide sample of lecturers, researchers, and professors: a pilot study (N = 120), a calibration study (N = 1084), and a validation study (N = 1481). Reliability and content, construct, and nomological validity were supported, as well as measurement invariance across work role (researchers, associate professors, and full professors) and gender. Evidence from the present study shows that the AQ@workT represents a useful and reliable tool to assist university management to enhance quality of life, to manage work-related stress, and to mitigate the potential for harm to academics, particularly during a pandemic. Future studies, such as longitudinal tests of the AQ@workT, should test predictive validity among the variables in the tool.

## 1. Introduction

In the present scenario, universities are characterized by high levels of work-related stress (WRS) in academics [1,2]. The rapid changes that academics have experienced over recent years are resulting in several challenges for those who work in higher education institutions [3]. These changes, which transversely affect all workers in universities, result in greater work intensification [4], which may have negative outcomes, including higher levels of work–home interference and turnover intentions [5]. Recent research suggests that academics are experiencing pressures at work from a variety of sources, including: the introduction of fixed-term contracts; rapid growth in student numbers; difficulties in seeking funding; competition with colleagues and role ambiguity; difficulties in working with the latest digital technologies; and politics within the workplace that have led teaching and research staff to be more exposed than ever to the risk of WRS and ill-health [6]. Moreover, studies across different cultural contexts have shown that rising job demands, both in quantity (e.g., work overload) and quality (e.g., increasing administrative demands), may negatively affect academics’ mental health and well-being; in turn, this may lead to excessive WRS, burnout, and mental health issues [7].

In addition, due to the COVID-19 pandemic, new teaching and learning modalities have given rise to a “new beginning” for students and for faculty members, who have had to abruptly shift from face-to-face teaching to fully online modalities. For example, faculty members had to quickly implement new digital teaching practices to promote students’ learning while maximizing their safety [8]. Although online teaching can have some positive outcomes, such as increased autonomy and flexibility in terms of workspace and time, as well as reduced commuting time, it can also lead to increased pressure on university academics due to the constant use of Information and Communication Technologies (ICT; e.g., online teaching and meetings) [9]. For academics, the integration of ICT into classrooms can result in work overload, role ambiguities, changed patterns of work, the need to constantly upgrade their digital knowledge and skills, and higher demands for performance and productivity [10]. Not surprisingly, previous research showed that working from home using ICT could generate feelings of tension, anxiety, exhaustion, and decreased job satisfaction [11,12]. Therefore, the need to be constantly productive may have implications for academics; on the one hand, accentuating multiple traditional risk factors and on the other, creating the emergence of new risks linked to the new methods, schedules, responsibilities, and demands for greater interaction with students and administrative staff. In this scenario, academics have become aware that the risks of WRS should be addressed, or prevented, and several movements in academia within the United Kingdom and Europe have been started to promote and enhance a culture of organizational well-being in academia (e.g., Healthy Universities https://healthyuniversities.ac.uk/ (accessed on 15 January 2022), or REMO—Research Mental Health Observatory—https://projects.tib.eu/remo/ (accessed on 15 January 2022)). In Italy, in 2016, a group of academics enrolled in the Italian Association of Psychologists (AIP) founded a research network named QoL@Work (Quality of Life at Work; https://aipass.org/qolwork-quality-life-work (accessed on 15 January 2022),). The aims of the group are: (a) to promote scientific comparisons of theoretical models, research methodologies, and measurement tools; (b) to propose guidelines and improvement actions within the academic context (i.e., university management); and (c) to create opportunities for exchanges with stakeholders (i.e., unions, higher education sector). As such, drawing on the Job Demands-Resources (JD-R) theoretical framework [13], the QoL@Work network engaged in adapting measures to assess the quality of life at work in academia to develop and validate a new tool (the AQ@workT), specifically addressed at investigating the well-being of researchers, lecturers, and professors. A related tool dedicated to administrative staff is currently being developed and will be reported in a subsequent work.

In view of the above, the present study aimed to introduce the AQ@workT and discuss its psychometric properties. The following section of the paper presents a brief literature review of the risks and protective factors of WRS in academia by adopting the JD-R model as a wider framework to study well-being and quality of life in the academic context. Furthermore, the process that led to the development of this tool will be detailed.

### 1.1. Risk and Protective Factors for Work-Related Stress in Academia

Findings from studies exploring the main factors that can lead to WRS or burnout among academics highlight the crucial role of workload demands, in terms of quantitative workloads, namely the amount of work to be done within a given time, as well as qualitative workloads, which relate to the difficulty or complexity of the job, particularly when the worker is not trained or does not have adequate resources to deal with their job role [14,15]. In academia, the former can reflect the increased number of courses that academics are required to develop and teach as well as overlapping tight deadlines [16], whereas the latter encompasses the number and diversity of tasks to be performed (e.g., teaching, research, and public engagement) [17]. Moreover, previous research has shown that (poor) interpersonal relationships—for example, with colleagues and students—may affect academics’ health and well-being [18,19,20]. Additional risk factors include job insecurity, especially for non-tenured faculty members [17,21], the increasing number of hours and demands for professional growth [22], difficulties in maintaining a healthy work–life balance [23], emotional labor [24], role overload, and role conflicts [18]. Finally, since academic work is “unusual” because it is characterized by a high intrinsic motivation for research and teaching activities [25], an increase in workload in terms of additional research or teaching commitments does not necessarily link to forms of dissatisfaction or discomfort, but should be monitored in terms of workaholism or exhaustion risks in the long term [25].

Beyond the aforementioned risk factors, some aspects of work in academia may prevent WRS (i.e., they are protective factors), as well as foster motivation and well-being. These primarily include social support from supervisors [26,27], co-workers [26,28], students [29], and administrative staff [30]. Other key protective factors are social recognition (e.g., the extent to which an academic’s work is recognized and appreciated by university management) [18], and job autonomy [31] in terms of control and freedom in teaching and undertaking research, which is associated with increased work-related motivation [27] and work–life balance [32]. Furthermore, opportunities for career advancement (e.g., institutional efforts to support the career development of faculty members), rewards (e.g., the distribution of rewards based on merit), as well as receiving feedback on one’s work, appear to positively influence academics’ mental health and job satisfaction [7]. According to the above literature and drawing upon the JD-R model [33], the QoL@Work network developed a conceptual framework that was reflected in a new assessment tool, the AQ@workT, tailored to professors and researchers in an academic context.

### 1.2. The Job Demands-Resources Model in Academia

The JD-R model was developed to provide a comprehensive framework to understand the factors that may challenge or enhance workers’ physical, mental, and psychological well-being. Since its first release in the early 2000s, the JD-R model has been significantly modified and extended; the first version of the model focused on burnout as a primary outcome variable, with later versions adding more diverse outcomes and antecedents, including personal and work resources. The core of the JD-R model identifies two categories of working conditions: job demands and job resources. Job demands are those “aspects of work that require prolonged physical or mental effort and are therefore associated with certain physiological conditions and psychological costs” (p. 501, [34]). Depleted energy and increased stress from responding to high job demands gradually leads employees to feel exhausted and tired, and to be emotionally exhausted. Therefore, high job demands are assumed to have a direct positive relationship with burnout, which is a chronic state of work-related stress characterized by emotional exhaustion (i.e., feeling emotionally drained and consumed), mental distancing (i.e., cynicism and lack of enthusiasm), and reduced personal effectiveness (i.e., doubting one’s competence and contribution to work) (p. 1, [35]). Burnout is a syndrome of chronic exhaustion in which a negative attitude to work leads to workers perceiving that they are less effective in their job [36]. Although job demands do not necessarily have a negative outcome, they can induce people to quit their job when the individual does not have the resources to satisfy those specific job demands [33], which can include high work pressure, heavy workload, time pressure, high levels of responsibility, and emotionally demanding interactions with clients.

Unlike demands, job resources refer “to the physical, social, psychological and organizational aspects of work which can be functional for the achievement of work objectives; able to reduce job requests and the physiological and psychological costs related to them; to stimulate personal growth, learning and development” (p. 2, [37]). Thus, job resources can provide workers with the support needed to accomplish goals and foster high levels of work commitment and may include autonomy, skill variety, performance feedback, and opportunities for growth [17]. Both these broad categories activate two different pathways: a health-impairment process and a motivational process. Specifically, the impairment process leads to energy depletion and negative work behaviors, such as burnout; conversely, the motivational process leads to the development of positive behaviors, such as work engagement, which is defined “as a positive, fulfilling, work-related state of mind and is characterized by vigor, dedication and absorption” (p. 295, [38]). “Vigor” refers to the worker having high levels of mental energy and resilience towards his or her work; and “dedication” is determined by a workers’ strong involvement in their job, which can be characterized by having a sense of meaning, enthusiasm, inspiration, pride, and challenge in their work. Finally, “absorption” refers to being totally focused and absorbed in one’s work to the point of not realizing how quickly time passes [38].

The addition of personal resources is an important extension of the JD-R model [39]. Personal resources refer to the worker’s positive self-assessment of their own abilities and the extent to which they believe that they have control over their organizational working environment, thereby predicting their job success, intrinsic motivation, and commitment to their work [40,41]. However, the successful integration of personal resources in the JD-R model depends on the type of personal resource being investigated; personal resources may act as antecedents (e.g., stable personality traits) of job demands and resources, or mediators (e.g., flexible traits) [42].

The JD-R model provides a simple but complete heuristic framework linking a wide variety of characteristics related to job content and a wide range of job results [43]. The model is flexible and easily adaptable to various organizational contexts and is currently the most accepted framework used in occupational health psychology to explore the relationships between job characteristics and employee well-being [44]. Several studies in academic settings across the world have adopted this theoretical framework to identify the job demands and resources that can decrease or increase an academics’ quality of life [16,28,45,46,47].

## 2. Methods

The purpose of this study is to describe the preliminary validation process of the AQ@workT, which was developed to assess the quality of life in lecturers, researchers, and professors within academia. The process of validating this new tool was undertaken in three studies. The first was a pilot study aiming to identify the core dimensions of the construct “quality of life at work in academia” and adapt and test the measures that were selected. The second study tested the new AQ@workT on a calibration sample. Finally, the third study retested the final version of the questionnaire on a validation sample.

### 2.1. Ethics

Before completing the AQ@workT, participants were asked to provide their informed consent and given information so that they would understand the aims of the study. Moreover, participants had the opportunity to withdraw from the study at any time. The research project was approved by the Ethical Board of the University of Bologna (Prot. 327010 del 19 December 2019), and data were organized and de-identified so that there was no possibility of tracing responses back to individual participants.

### 2.2. Research Structure and Data Analysis

As mentioned above, three studies were conducted to provide evidence of the psychometric properties of the AQ@workT.

In the pilot study (Study I), the aims were: the identification of the core dimensions of the tool; the adaptation of the scales to the academic context; and preliminary analyses of its psychometric properties. Scale reliability was assessed computing Cronbach’s alpha, whereas the dimensionality of the tool was explored with exploratory factor analysis (EFA). To enhance content validity, in terms of the choice of the core dimensions, and construct validity regarding the wording of the items, focus groups with subject-matter experts were conducted.

In the calibration study (Study II), after investigating the sample distribution of the data to verify whether all scales had a range of skewness and kurtosis values from −2 to +2 and from −7 to +7, respectively [48], the factor structure of the scales, to explore construct validity, was determined using confirmatory factor analysis (hereafter CFA) with MPlus software, version 8.5. CFAs were performed by aggregating the various latent constructs into demands, resources and mediators following the theoretical framework of the JD-R model, while outcomes were explored individually. In particular, with regard to CFAs, the following were considered as appropriate indices: comparative fit index (hereafter CFI) ≥ 0.90, root mean square error of approximation (hereafter RMSEA) ≤ 0.08, and standardized root mean square residuals (hereafter SRMR) ≤ 0.10 as threshold values [49]. Reliability was also measured using McDonald’s omega index.

In the validation study (Study III), the analyses conducted in Study II were replicated and nomological validity was assessed with a correlation analysis among the latent variables. Finally, measurement invariance analyses were performed considering gender (male, female) and academic role (researchers, associate professors, and full professors) as classification variables. To verify whether the invariance was respected, multigroup CFAs testing separate nested CFA models were conducted. First, the configural invariance model, in which all the parameters were freely estimated, was tested. Then, the metric invariance model, where invariant factor loadings met requirements, was examined. Finally, the scalar invariance model and the invariant intercepts were inspected. Finally, the models looking for differences in CFI, RMSEA, and SRMR were assessed. Chen’s [50] suggestion was followed, so that a change in CFI less or equal to 0.010, a change in RMSEA less or equal to 0.015, and a change in SRMR less or equal to 0.030 was considered as the threshold for testing metric invariance, and less or equal to 0.010 for assessing scalar invariance.

## 3. Study I: Identification of the Core Dimensions of the Tool

In Study 1, existing scales relating to quality of life at work, and specifically to academics, were collected from the literature and used as the initial foundation of the questionnaire. Using the framework of the JD-R model, the QoL@Work network reviewed the literature and identified a large set of variables.

**Figure 1:**
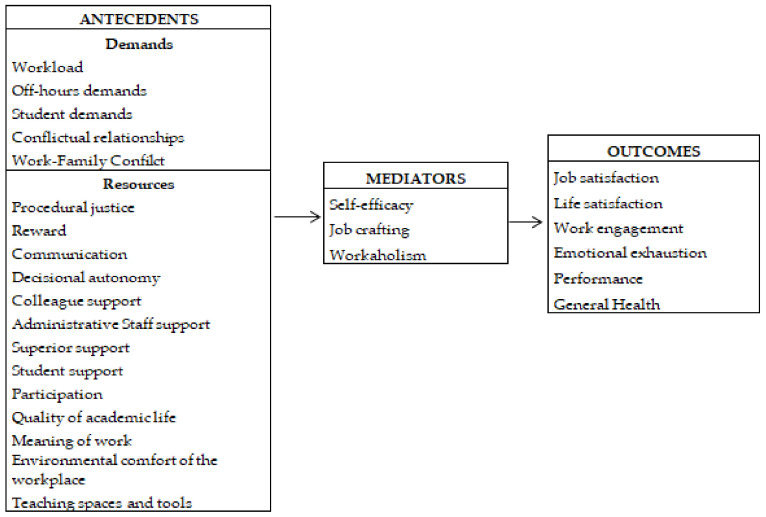
Set of variables composed of demands, resources, mediators, and outcomes included in Study 1.

Ten focus groups were conducted by five members of the QoL@Work Network with subject-matter experts in their own academic context. The focus group participants were individuals who were members of the local risk assessment group at each university. The Legislative Decree 81/2008 in Italy established the obligation to assess WRS in all Public and Private Administrations. The Italian guidelines for risk assessment require a group composed of 5–6 members for each organization, and must include an occupational doctor, staff representatives, and experts in the field of safety and health in the workplace. In each university, a focus group with 5/6 participants (members of the risk assessment working group) was organized with the aim of collecting possible suggestions, observations, and critical issues. Subsequently, a national focus group was formed (N = 22), composed of representatives from Italian academia (members of QoL@Work), to develop and identify the most important variables in the current literature based upon the focus group participants’ suggestions. Finally, based on the insights and experience of participants in the focus groups, and bearing in mind contextual and geographical characteristics, the principal risk and protective factors for stress in academic settings were identified. Once these procedures were completed, a set of variables was chosen and used to create the first version of the questionnaire (Figure 1). This instrument comprised a series of variables that was divided into demands, resources, moderators/mediators, and outcomes in accordance with the JD-R theoretical model. The questionnaire was composed of 159 items. A final section of the questionnaire included a question in order to analyze the extent and clarity of the information requested and if there were any other issues related to the questionnaire (i.e., length, words used). In a further step, the psychometric properties of the resulting instrument were then assessed.

### 3.1. Procedure and Participants

An online pilot survey was conducted with 152 Italian academics (lecturers, researchers, and professors) who were selected using avalanche sampling. Members of the QoL@Work network personally contacted some of their colleagues who were interested in participating in the pilot survey. After deleting those with more than 15% of missing data (to avoid influencing the analyses with high-value data estimations), the final sample comprised 120 participants who were predominantly female (58.3%) and had a mean age of 49.7 years (SD = 7.8), with a range from 36 to 69 years. Missing data of less than 15% were dealt with using the full information maximum likelihood (FIML) algorithm.

### 3.2. Results Study 1

After preliminary analyses on missing distribution and normality assumptions, reliability analyses were assessed using Cronbach’s alpha, and the dimensionality of the tool was explored with an EFA. All scales showed good reliability, ranging from 0.72 to 0.95, except the scale on the environmental comfort of the workplace (alpha = 0.67). EFA confirmed the factor structure of the scales highlighting a good rate of explained variance, ranging from 41 to 87%. Thirty percent of respondents expressed doubts about aspects such as questionnaire length, privacy, the use of some scales, and the wording used. The average time taken to complete the questionnaire was about 35 min.

After the pilot study, and to take into account any critical issues raised by respondents, the questionnaire was revised and re-evaluated through focus groups by subject-matter experts from the QoL@Work network who, on the basis of the literature and the variables used, finalized the AQ@workT. In particular, as some respondents commented that the questionnaire was too long, the experts discussed the most suitable way to reduce the length of the tool and agreed to include only the most significant and suitable dimensions for assessing quality of life and WRS in the academic context within the tool. In view of the above, some of the variables were removed from the tool (e.g., meaning of work from the resources category, job crafting from the moderator/mediator category, and general health and performance from the outcomes).

The final version of the tool was then analyzed further in two additional studies to verify its psychometric characteristics and measurement invariance.

## 4. Study II: Analyses of the Psychometric Properties of the AQ@workT in a Calibration Sample

### 4.1. Procedure and Participants

The next step involved further phases of analyses undertaken in a sample of five Italian universities in different regions (two from northern Italy and three from southern Italy). In order to obtain participants, all teaching staff at the five universities were sent an invitation email. The obtained dataset was randomly divided into two groups, one used for the calibration and the other for the validation analyses (see next paragraph). According to the procedure used for all studies (i.e., eliminating observations with more than 15% of missing data and estimating the others using FIML), the two samples included 1084 and 1481 subjects, respectively. Therefore, the final sample for the calibration study was composed of 1084 university lecturers, comprising 32.9% researchers (both permanent and fixed-term), 43.7% associate professors, and 23.4% full professors. There was a slight majority of men (54.0%) and only 0.3% of respondents were under 30 years old, 53.0% were between 31 and 50 years old, and the remaining 46.7% were over 51 years old. This calibration sample enabled the preliminarily investigation of the main psychometric characteristics of the tool’s core dimensions, identified after adapting the items to the specific context according to the suggestions and issues elicited by the previous pilot study (Study 1). Furthermore, construct validity of the scales selected via the abovementioned procedure for the pilot study was assessed through CFAs to explore the factorial structure of the tool. Reliability analysis was used to verify items’ internal consistency. The items that did not enable the robustness of the scales to be validated were deleted from the analysis after determining that they were not core from the point of view of construct meaning. Moreover, missing data of less than 15% were estimated using the FIML algorithm, while observations with higher missing ratios were removed.

### 4.2. Measures

**Figure 2:**
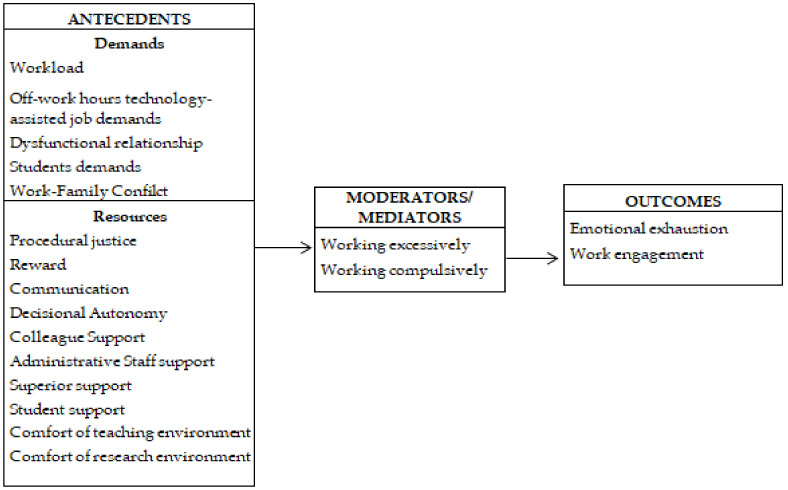
Set of variables composed of demands, resources, mediators, and outcomes included in Study 2.

The final version of the tool was composed of 72 items (see Table A1), classified according to the JD-R framework of demands, resources, moderators/mediators and outcomes, as reported in Figure 2.

### 4.3. Job Demands

Workload and academic workload, three items from Boyd et al. [51], for example: “I don’t have enough time to do quality research”, and three items from Edwards et al. [52], Toderi et al., [53] and Melin et al. [54], for example: “I have unreachable deadlines” with a response scale from 1 = Totally disagree to 6 = Totally agree.

Off-work hours (technologically assisted job demands), two items from Ghislieri et al. [55], for example: “I find myself answering the telephone or emails outside working hours”, with a response scale from 1 = Never or almost never to 6 = Always or almost always.

Dysfunctional relationships, four items from the Stress Indicator Tool [52,53] and Q-Bo test [56], e.g., “There is friction or conflict between colleagues”, with a response scale from 1 = Totally disagree to 6 = Totally agree.

Excessive student demands, four items adapted from customer-related social stressors [57], e.g., “Students make excessive demands”. The response scale is from 1 = Never or almost never to 6 = Always or almost always.

Work–family conflict, three items from Carlson et al. [58], e.g., “My work keeps me from my family activities more than I would like”, with a response scale from 1 = Totally disagree to 6 = Totally agree.

### 4.4. Job Resources

Procedural justice, three items from Colquitt [59] and Spagnoli et al. [60] e.g., “The procedures used to allocate resources in the department were applied with seriousness and reliability”. Response scale from 1 = Never or almost never to 6 = Always or almost always.

Reward, four items created by the research team’s subject matter experts, e.g., “Express how rewarded you feel: In the positions I hold at institutional and/or departmental level”, with a response scale from 1 = Not at all to 6 = All rewarded.

Quality of communication, two items from the Copenhagen Psychosocial Questionnaire [61], e.g., “I am informed in good time regarding changes, plans for the future, important decisions”. Response scale from 1 = Totally disagree to 6 = Totally agree.

Decisional autonomy, three items from De Carlo et al. [56], e.g., “My job allows me to decide with a certain degree of autonomy on the programming and planning of the activities I carry out”. Response scale from 1 = Totally disagree to 6 = Totally agree.

Colleagues’ support, four items from the Stress Indicator Tool [52,53], e.g., “Colleagues give me the help and support I need”, with a response scale from 1 = Totally disagree to 6 = Totally agree.

Support from administrative staff, three items created by the team’s subject matter experts, e.g., “Please indicate how supported you feel regarding: accounting aspects”, with a response scale from 1 = Never to 6 = Always.

Hierarchical superiors’ support, three items from Balducci et al. [62], e.g., “I receive support information from my Head of Dept. who helps me in the work I do”. Response scale from 1 = Never or almost never to 6 = Always or almost always.

Students’ support, four items from the Customer-Initiated Support scale [29,63], e.g., “Students are on the same wavelength as me”, with a response scale from 1 = Never or almost never to 6 = Always or almost always.

Participation, 3 items adapted from the Job Content Questionnaire [64], e.g., “In my department, I have influence on decisions that affect my scientific sector”, with a response scale from 1 = Never to 6 = Always.

Comfort of teaching environments, two items created by the team’s subject matter experts, e.g., “Assess the level of appropriateness of the following aspects of your working environment: The state of the teaching facilities”. Response scale from 1 = Not completely appropriate to 6 = Completely appropriate.

Comfort of research environments, two items created by the team’s subject matter experts, e.g., “Assess the level of appropriateness of the following aspects of your working environment: The state of the research facilities”, with a response scale from 1 = Not completely appropriate to 6 = Completely appropriate.

### 4.5. Hypothesized Moderator/Mediator Variables

Workaholism was assessed by the two subdimension of the Dutch Work Addiction Scale (DUWAS) adapted in Italy by Balducci et al. [62]:

Working excessively, five items, e.g., “I seem to be in a hurry and racing against the clock”. Response scales from 1 = Never, almost never to 6 = Always, almost always.

Working compulsively, five items, e.g., “I feel that there’s something inside me that drives me to work hard”. Response scales from 1 = Never, almost never to 6 = Always, almost always.

### 4.6. Outcome Variables

Work engagement, six items from Balducci et al. [65], e.g., “In my work I feel full of energy” were used.

Emotional exhaustion, five items from Kristensen et al. [61], e.g., “I feel exhausted at the end of a workday” were used.

Both these outcome measures had a response scale ranging from 1 = Never, almost never to 6 = Always, almost always.

### 4.7. Results Study 2

Sample distribution, descriptive statistics, and measurement reliability (through McDonald’s omega index) of the calibration sample are presented in Table 1.

Mean values for asymmetry and kurtosis for all the scales are within the range −2/+2 and −7/+7, respectively, supporting normality [48]. Regarding the descriptive statistics, it is of interest to highlight some results. For example, among job demands, Workload and Off-work hours technology-assisted job demands had the higher means; whilst in the resources area, Participation showed the lowest mean and Decisional Autonomy and Students’ support the highest; for the mediators, Working Excessively had the highest mean; and with regard to outcomes, the mean for Work Engagement was the highest.

McDonald’s omega values highlighted good reliability for all the scales, except for ‘Workload’, which showed a lower level of reliability (0.66).

Furthermore, regarding confirmatory factor analyses, the proposed aggregations into demands, resources, mediators, and outcomes showed good fit indices and all factor loadings were statistically significant, as highlighted in Table 2.

## 5. Study III: Validation of Measurement Scales and Measurement Invariance

### 5.1. Procedure and Participants

Results from CFAs conducted on the calibration sample led to a further study involving 1481 university researchers/professors, determined, as mentioned above, by randomly partitioning the total sample. As in Study 2, the sample was recruited by directly sending a participation link to all the teaching staff of the five universities. Researchers accounted for 38.5% of the sample, associate professors for 41.7%, and full professors for 19.8%. Once again, there was a slight majority of men (56.0%), and 50.4% of the sampled staff were aged between 31 and 50 years with the remainder (49.6%) being over 50 years of age. All participants worked at Italian universities (with two universities located in the north of Italy and three located in southern Italy). Missing data of less than 15% were handled by FIML estimation, while observations with more than this ratio were deleted in order to avoid non-real data bias.

### 5.2. Results Study 3

The results of the preliminary analyses of Study 2 were confirmed in this validation sample. Mean values for asymmetry and kurtosis for all the scales were within the range of −2/+2 and −7/+7, respectively, supporting normality [48]. The descriptive statistics highlighted the same trends found in the calibration sample (Study 2). In the job demands category, Workload and Off-work hours technology-assisted job demands again had the highest means; in the job resources area, Participation had the lowest mean, and Decisional Autonomy and Students’ Support had the highest means. Regarding mediators, Working Excessively was the highest, and in the outcomes area, the level of Work Engagement was highest, as reported in Table 3.

Reliability analyses showed that all constructs provided excellent McDonald’s omega indices, greater than 0.70. The nomological validity, which refers to the degree to which relationships in a formal theoretical network containing the constructs of interest are confirmed, was performed by a correlation analysis. Correlations of the latent variables confirmed those reported in the literature regarding the JD-R model, as stated in Table 4. Specifically, the scales that were categorized as job resources (i.e., procedural justice, reward, decisional autonomy) correlated positively with each other, and negatively with the job demands of workload and work–family conflict. These scales also correlated positively with outcomes related to the motivational process, such as work engagement, and negatively with the consequences of health impairment mechanisms, such as emotional exhaustion. Conversely, work demands correlated positively with each other and with processes related to energy reduction (emotional exhaustion), and negatively with work resources and work-related outcomes, such as work engagement. Interestingly, the perceived quality of the workplace (both teaching and research) correlates positively to many of the “social” job resource factors (e.g., justice, rewards, communication, support) and negatively to factors such as workload or exhaustion. Therefore, construct validity of all the measures used is supported.

Additionally, in this case, CFAs confirmed the factor structure of the tool, highlighting its construct validity (see Table 5). Accordingly, all factor loadings were statistically significant.

The measurement invariance, both metric and scalar, across gender and role was mostly confirmed for all the constructs (see Table 6 and Table 7). There were two exceptions: in the analyses considering metric invariance across gender, for the mediators, Δ CFI was slightly larger than 0.010 even if Δ RMSEA and Δ SRMR were lower than the cut-off; with regard to metric invariance across academic job roles, the Emotional exhaustion Δ RMSEA was larger than the cut-off, even if Δ CFI and Δ SRMR were smaller.

## 6. Discussion

The three studies conducted and presented in this paper (pilot, calibration, and validation study) supported the validation of the AQ@workT, a new tool developed by the Italian QoL@Work network to assess academic quality of life at work for lecturers, professors, and researchers. By adopting the JD-R model [33] as a framework, the AQ@workT presented satisfactory psychometric properties (normality of the items, reliability, and content, construct and nomological validity) and measurement invariance across gender and academic work role, indicating that the tool is a reliable and valid instrument to assess job demands, job resources, mediators, and outcomes in the working life of academics within Italian universities.

Although there is literature on WRS in academia, most of the current research is largely based on WRS models, such as the effort–reward imbalance (ERI) model [66] and the JD-R model [13], but appears incomplete because often, with few exceptions [46], it lacks a clear focus on the specific job profile of researchers and professors. One of the exceptions in this vein is the initial validation of a 24-item scale measuring the context-specific features of academic work and environments that characterize academics’ quality of life at work, namely the Academics’ Quality of Life at Work Scale (AQoLW) developed by Converso and colleagues [46]. This scale was tailored to assess the increasingly demanding academic environment and to understand if academics perceive several dimensions related to their work as challenges or as hindrance demands. We believe that the AqoLW could represent a useful measure for studying the specific academic context in depth as it could be integrated into a more comprehensive tool. In this perspective, the present paper contributes to this debate by presenting a new tool, the AQ@workT, aimed at incorporating the two main tenets (health impairment; motivation) of the JD-R model and expanding the model to understand the role played by psychosocial aspects of university workplaces in workers’ well-being, particularly during the COVID-19 pandemic. Psychosocial factors refer to organizational and/or work aspects (e.g., the use of digital technologies and teaching platforms) and interpersonal relationships (with colleagues, supervisors, students) in the work setting that may affect the health of workers [67]. For example, the job demands (i.e., challenges) and job resources (i.e., benefits) of digital technology platforms and their evolution and growth are now necessary for universities to survive and thrive within the higher education context, but their impact on the well-being of academics is not fully understood and requires investigation [68].

The AQ@workT, after undertaking cross-cultural measurement invariance analysis, could be applied in higher education sectors across the world as its core variables apply to the current working lives of academics who report high levels of WRS in various countries, including European countries, the United Kingdom [1], and Australia [47]. For example, the importance and relevance of the AQ@workT is supported by recent research by Wray and Kinman [2] who surveyed 2046 academic workers in the United Kingdom during the COVID-19 pandemic to investigate their mental health and well-being. By employing the Health Safety Executive’s (HSE) framework of work hazards, the researchers found that the academic cohort reported lower levels of well-being than the norms of the working population regarding demands, support from managers and colleagues, working relationships, and role clarity. Indeed, these levels were so low that they were identified as requiring urgent action. Furthermore, in relation to job demands, 79% reported that they needed to work very intensively often or always, and 52% of the sample often or always experienced unrealistic time pressures. Thus, it is not surprising that more than half of the sample (53%) showed signs of probable depression.

As the above-mentioned variables are part of the AQ@workT, the present study, together with recent evidence coming from the academic context, points to a critical issue in academia at an international level and clearly shows that there is much more that needs to be done to address the mental health, well-being, and quality of life of this professional group, particularly as COVID-19 has further increased the demands on academics and led to further deterioration in their quality of life [2]. Given that digital communication and digital platforms are fundamental to remote learning and now underpin teaching in universities across the globe, their use and influence will only grow and become more prominent in the future [69]; this will further increase the cognitive demands and stress levels of academics, who will need to constantly upgrade their knowledge and skills to meet higher demands for performance and productivity [10].

Although the positive results of our research support the good psychometric properties of the AQ@workT, the three studies presented have some limitations. First, these studies were cross-sectional in nature and thus, the direction of the associations between the variables, which is the criterion validity of the model, cannot be examined. Future validation studies should employ a longitudinal design to test the predictive relationship among the variables in the study. A longitudinal design would also allow a test of the longitudinal measurement invariance, to examine whether the same constructs are measured equally at different time points within the same group to ensure that any change in the observed scores over time can be attributed to actual development and/or changes in the construct under investigation [70].

Although the sampling in the present study was quite broad, another limitation is that the samples are not representative of the whole Italian academic population. Thus, present results cannot be generalized and future studies should include representative samples. Furthermore, it seems appropriate to increase our efforts to develop a version of the tool that can also be administered to the administrative staff working in academia, in order to establish common intervention themes and improve professional collaboration, well-being, and the effectiveness of university processes.

Another important limitation is related to the theme of cultural invariance. In this respect, as the tool was tested in Italy on Italian academic staff, the cross-cultural effectiveness of the instrument should be measured as part of further studies to ensure cultural invariance.

Moreover, future studies addressing the validation of the AQ@workT should also identify norm values, reporting the score distribution of the tool in a representative sample of the academic population, thereby providing the standard frame with which to compare the tool scores [71].

Finally, although the current “core” model of the tool is composed of variables conceived as critical for assessing the quality of life at work in academia within the current Italian university context, future studies should monitor the integration of further specific context-related scales, such as the AQoLW proposed by Converso and colleagues [46]. In this vein, we have already included that scale in future data collection so that we can provide a more comprehensive tool.

Future studies should also monitor the changes that may occur in academics’ roles, which can then be incorporated into this new tool. Likewise, the role of specific environmental elements that promote (and not merely permit) psychological restoration in the workplace (e.g., accessible outdoor green spaces or indoor plants), should also be more deeply investigated and incorporated in conceptual models, based on the increasing evidence linking restorative environments and human health [72].

## 7. Conclusions

The AQ@workT is a valuable and timely tool for investigating the extent of WRS in academics within the Italian university sector, and can also be applied to other knowledge workers in higher education sectors and similar industries both nationally and internationally. The tool was developed by a team of academics, experts in work and organizational psychology, and then validated in further calibration and validation samples that demonstrate the tool’s relevance and importance. The findings highlight a need for more evaluation, such as longitudinal tests to examine predictive relationships. The tool could be helpful to assist university management to enhance the quality of life and manage WRS and the ensuing and growing risk of harm, particularly during a pandemic. The value of integrating such knowledge is vital for enabling senior management to design and promote best practices for academics to manage and promote mental health and well-being within the global university sector.

## Figures and Tables

**Table 1:** Principal descriptive analysis of the calibration sample (N = 1084).

	N° of Items	Mean	DS	Reliability	Skewness	Kurtosis
DEMANDS						
Workload	6	3.79	0.95	0.66	0.40	0.78
Off-work hours technology-assisted job demands	2	4.86	1.40	0.89	1.26	0.51
Dysfunctional relationship	4	2.47	1.21	0.87	1.05	1.27
Students’ demands	4	2.23	1.07	0.87	1.07	0.94
Work–family conflict	3	3.74	1.56	0.95	0.13	1.18
RESOURCES						
Procedural justice	3	3.71	1.54	0.94	0.14	1.10
Reward	4	3.59	1.17	0.82	0.20	0.86
Communication	2	3.49	1.28	0.83	0.04	0.85
Decisional autonomy	3	4.53	1.15	0.89	0.78	0.28
Colleagues support	4	3.05	1.06	0.92	0.26	0.76
Administrative staff support	3	3.40	1.23	0.73	0.09	1.05
Superior support	3	3.86	1.61	0.92	0.29	1.21
Students’ support	4	4.33	1.13	0.87	0.65	0.27
Participation	3	2.74	1.47	0.92	0.56	0.82
Comfort of teaching environment	2	3.37	1.42	0.91	0.05	1.07
Comfort of research environment	2	3.18	1.38	0.84	0.14	1.01
MODERATORS/MEDIATORS						
Working excessively	5	4.63	1.05	0.85	0.90	0.71
Working compulsively	5	3.77	1.11	0.78	0.41	0.81
OUTCOMES						
Emotional exhaustion	5	2.82	1.28	0.87	0.65	0.64
Work engagement	6	4.27	1.11	0.91	0.86	0.40

**Table 2:** Confirmatory factor analysis aggregated by demands, resources, mediators, and outcomes in calibration sample (N = 1084).

Calibration Sample				
Models	CHI (DF)	CFI	RMSEA	SRMR
Job demands	747.29 (174)	0.947	0.046 (0.042–0.050)	0.046
Job resources	2563.86 (440)	0.924	0.057 (0.055–0.059)	0.061
Mediators/Moderators	322.47 (33)	0.948	0.077 (0.069–0.085)	0.053
Emotional exhaustion	69.24 (5)	0.963	0.095 (0.076–0.116)	0.028
Work engagement	66.91 (7)	0.981	0.076 (0.060–0.093)	0.021

**Table 3:** Principal descriptive analysis of the validation sample (N = 1481).

	N° Item	Mean	DS	Reliability	Skewness	Kurtosis
DEMANDS						
Workload	6	3.77	0.95	0.71	0.47	0.88
Off-work hours technology-assisted job demands	2	4.74	1.42	0.89	1.04	0.32
Dysfunctional relationship	4	2.53	1.30	0.88	0.95	1.18
Students’ demands	4	3.47	1.26	0.83	1.02	0.79
Work–family conflict	3	3.62	1.55	0.95	0.15	1.14
RESOURCES						
Procedural justice	3	3.55	1.48	0.94	0.10	1.07
Reward	4	3.59	1.23	0.80	0.14	1.00
Communication	2	3.44	1.33	0.85	0.04	0.93
Decisional autonomy	3	4.51	1.13	0.86	0.71	0.26
Colleagues support	4	3.21	1.22	0.92	0.17	0.90
Administrative staff support	3	3.47	1.26	0.73	0.06	1.10
Superior support	3	3.76	1.62	0.89	0.21	1.28
Students’ support	3	3.83	1.37	0.89	0.28	0.92
Participation	3	2.95	1.54	0.92	0.37	1.13
Comfort of teaching environment	2	3.58	1.45	0.90	0.10	1.05
Comfort of research environment	2	3.46	1.36	0.82	0.04	0.95
MEDIATORS						
Working excessively	5	4.61	1.06	0.86	0.85	0.53
Working compulsively	5	3.82	1.11	0.79	0.41	0.77
OUTCOMES						
Emotional exhaustion	5	2.70	1.18	0.84	0.71	0.65
Work engagement	6	3.92	1.33	0.94	0.40	0.85

**Table 4:** Correlation matrix between latent variables on the validation sample.

	1	2	3	4	5	6	7	8	9	10	11	12	13	14	15	16	17	18	19	20	21
1. Academic workload	-																				
2. Workload	0.88 ***	-																			
3. Off-work hours technology-assisted job demands	0.29 ***	0.52 ***	-																		
4. Conflictual relationship	0.16 **	0.29 ***	0.15 ***	-																	
5. Students’ demands	0.33 ***	0.29 ***	0.16 ***	0.15 ***	-																
6. Work–family conflict	0.44 ***	0.66 ***	0.46 ***	0.20 ***	0.21 ***	-															
7. Procedural justice	−0.06	0.00	0.02	−0.34 ***	−0.16 ***	-0.04	-														
8. Reward	−0.33 ***	−0.13 **	0.07	−0.22 ***	−0.11 **	−0.11 **	0.36 ***	-													
9. Communication	−0.18 **	−0.12 **	−0.06	−0.24 ***	−0.12 ***	−0.09 **	0.59 ***	0.45 ***	-												
10. Decisional autonomy	−0.43 ***	−0.30 ***	−0.12 **	−0.18 ***	−0.14 **	−0.30 ***	0.22 ***	0.39 ***	0.25 ***	-											
11. Colleagues support	−0.15 **	−0.12 ***	−0.04	−0.24 ***	−0.11 **	−0.15 ***	0.41 ***	0.51 ***	0.48 ***	0.26 ***	-										
12. Administrative staff support	−0.34 ***	−0.13 **	−0.06	−0.16 ***	−0.11 **	−0.06	0.46 ***	0.42 ***	0.52 ***	0.19 ***	0.45 ***	-									
13. Superior support	−0.12	−0.06	−0.03	−0.24 ***	−0.03	−0.09 **	0.58 ***	0.51 ***	0.50 ***	0.25 ***	0.50 ***	0.45 ***	-								
14. Students’ support	−0.17	0.03	0.06	−0.08 **	−0.15 ***	−0.03	0.02	0.42 ***	0.00	0.16 **	0.09 **	0.04	0.24 ***	-							
15. Participation	0.02	−0.04	−0.02	−0.13 ***	−0.07 **	−0.06	0.44 ***	0.37 ***	0.49 ***	0.23 ***	0.38 ***	0.27 ***	0.34 ***	−0.17 ***	-						
16. Comfort of teaching environment	−0.19 **	−0.12 ***	-0.06 **	−0.04	−0.14 ***	−0.08	0.28 ***	0.15 ***	0.31 ***	0.13 **	0.18 ***	0.38 ***	0.11 ***	−0.09 **	0.22 ***	-					
17. Comfort of research environment	−0.27 **	−0.14 **	−0.06	−0.06 **	−0.10 **	−0.11 **	0.34 ***	0.27 ***	0.39 ***	0.21 ***	0.27 ***	0.43 ***	0.19 ***	−0.10 **	0.32 ***	0.65 ***	-				
18. Working excessively	0.55 ***	0.79 ***	0.55 ***	0.21 ***	0.18 ***	0.62 ***	0.03	−0.07	−0.09 **	−0.21 ***	−0.05	−0.09 **	−0.03	0.02	−0.04	−0.07 **	−0.10 **	-			
19. Working compulsively	0.39 ***	0.44 ***	0.32 ***	0.16 ***	0.17 ***	0.44 ***	0.01	−0.05	0.01	−0.23 ***	−0.02	0.02	0.00	0.00	−0.01	−0.04	−0.07 **	0.63 ***	-		
20. Emotional exhaustion	0.40 ***	0.52 ***	0.23 ***	0.41 ***	0.30 ***	0.43 ***	−0.17 ***	−0.29 ***	−0.15 ***	−0.35 ***	−0.18 ***	−0.14 ***	−0.17 ***	−0.14 ***	−0.07 **	−0.09 **	−0.13 ***	0.46 ***	0.43 ***	-	
21. Work engagement	−0.32 ***	−0.06	0.06 **	−0.17 ***	−0.06	−0.10	0.14 **	0.57 ***	0.19 ***	0.41 ***	0.30 ***	0.19 ***	0.39 ***	0.61 ***	−0.07 **	−0.04	−0.01	0.00	0.00	−0.35 ***	-

Note: *** *p* value < 0.001, ** *p* value < 0.05.

**Table 5:** Confirmatory factor analysis aggregated by demands, resources, mediators, and outcomes in validation sample (N = 1481).

Validation Sample				
Models	CHI (DF)	CFI	RMSEA	SRMR
Job demands	632.05 (174)	0.935	0.049 (0.045–0.053)	0.067
Job resources	1431.87 (440)	0.946	0.046 (0.043–0.048)	0.064
Mediators	296.75 (32)	0.936	0.089 (0.080–0.098)	0.060
Emotional exhaustion	73.13 (5)	0.953	0.115 (0.093–0.140)	0.031
Work engagement	7.20 (7)	1	0.005 (0.000–0.038)	0.025

**Table 6:** Results of invariance analyses for demands and resources across gender (male, female) and across academic roles (researcher, associate professor, full professor).

Constructs Groups Model	*χ2(df)*	CFI	RMSEA	SRMR	Δ CFI	Δ RMSEA	Δ SRMR
Demands Gender Configural inv.	790.10(348)	0.945	0.045	0.055	–	–	–
Metric inv.	833.82(363)	0.942	0.047	0.058	0.002	0.002	0.003
Scalar inv.	872.16(378)	0.939	0.047	0.059	0.003	0.000	0.001
Role Configural inv.	1221.92(522)	0.930	0.052	0.067	–	–	–
Metric inv.	1264.24(552)	0.929	0.051	0.070	0.001	0.001	0.003
Scalar inv.	1338.31(582)	0.925	0.051	0.071	0.004	0.000	0.001
Resources Gender Configural inv.	3037.13(880)	0.923	0.058	0.064	–	–	–
Metric inv.	3053.98(902)	0.923	0.057	0.064	0.000	0.001	0.000
Scalar inv.	3106.93(924)	0.922	0.057	0.064	0.001	0.000	0.000
Role Configural inv.	3738.66(1320)	0.914	0.061	0.071			
Metric inv.	3781.86(1364)	0.914	0.060	0.071	0.000	0.001	0.000
Scalar inv.	3897.23(1408)	0.911	0.060	0.071	0.003	0.000	0.000

Note. df = degrees of freedom; CFI = comparative fit index; RSMEA = root-mean-square error of approximation; SRMR = standardized root-mean-square residual; Δ CFI/RSMEA/SRMR = change in CFI/RSMEA/SRMR.

**Table 7:** Results of invariance analyses for mediators and each output construct across gender (male, female) and across academic role (researcher, associate professor, full professor).

Constructs	Groups	Model	*χ2(df)*	CFI	RMSEA	SRMR	Δ CFI	Δ RMSEA	ΔSRMR
Mediators	Gender	Configural inv	388.088(66)	0.942	0.081	0.057	–	–	–
		Metric inv.	404.95(74)	0.940	0.078	0.063	0.002	0.003	0.006
		Scalar inv.	489.60(82)	0.926	0.082	0.071	0.014	0.004	0.008
	Role	Configural inv.	404.73(99)	0.945	0.079	0.056	–	–	–
		Metric inv.	429.26(115)	0.940	0.074	0.064	0.005	0.005	0.008
		Scalar inv.	474.31(131)	0.938	0.073	0.070	0.002	0.001	0.006
Emotional Ex.	Gender	Configural inv	83.74(10)	0.957	0.102	0.031	–	–	–
		Metric inv.	94.31(14)	0.954	0.090	0.040	0.000	0.001	0.000
		Scalar inv.	109.04(18)	0.947	0.084	0.048	0.007	0.006	0.008
	Role	Configural inv.	87.98(15)	0.958	0.101	0.031	–	–	–
		Metric inv.	100.61(23)	0.956	0.084	0.039	0.002	0.017	0.008
		Scalar inv.	117.25(31)	0.951	0.077	0.041	0.002	0.007	0.003
Work Engag.	Gender	Configural inv	76.52(14)	0.981	0.078	0.022	–	–	–
		Metric inv.	84.90(18)	0.980	0.071	0.023	0.001	0.007	0.001
		Scalar inv.	97.01(22)	0.977	0.068	0.023	0.003	0.003	0.000
	Role	Configural inv.	82.11(21)	0.982	0.077	0.022	–	–	–
		Metric inv.	103.62(29)	0.978	0.072	0.039	0.004	0.005	0.017
		Scalar inv.	121.06(37)	0.975	0.068	0.042	0.003	0.004	0.003

Note. df = degrees of freedom; CFI = comparative fit index; RSMEA = root-mean-square error of approximation; SRMR = standardized root-mean-square residual; Δ CFI/RSMEA/SRMR = change in CFI/RSMEA/SRMR.

## Data Availability

The data presented in this study are available on request from the corresponding author. The data are not publicly available due to privacy reasons.

## Appendix A

**Table A1:** The final 72 items of the AQ@workT scale.

Job Demands	
Workload	I don’t have enough time to do quality research
	2. The number of hours I devote to teaching has increased or is excessive
	3. The load of administrative tasks I am required to perform can be managed
	4. I have unreachable deadlines
	5. I have to work very hard
	6. I have to neglect certain tasks because I have too much to do
Off-work hours technology-assisted job demands	I find myself answering the telephone or emails outside working hours
	2. I find myself answering the phone or emails during holidays
Dysfunctional relationships	I am subject to bullying or harassment at work
	2. At work I am subject to personal harassment in the form of rude words and behavior
	3. There is friction or conflict between colleagues
	4. Workplace relationships are strained
Excessive students’ demands	Students make excessive demands
	2. Students make my work worse because they are not motivated or interested
	3. Students complicate my work because they do not follow the rules
	4. Students burden my work by making improper demands
Work–family conflict	My work keeps me away from my family life more than I would like
	2. The time I have to devote to my work prevents me from participating as much as I would like in family life
	3. I cannot participate in family activities because of the amount of time required by my job
Job resources	
Procedural justice	The procedures used to allocate resources in the department were applied with seriousness and reliability
	2. The procedures used to allocate resources in the department were applied unprejudiced.
	3. The procedures used to allocate resources in the department were based on highly precise information
Reward	Express how rewarded you feel: In the positions I hold at institutional and/or departmental level
	2. Express how rewarded you feel: In teaching activities
	3. Express how rewarded you feel: In third mission activities
	4. Express how rewarded you feel: In research activities
Quality of communication	I am informed in good time regarding changes, plans for the future, important decisions
	2. It is easy to get the information I need
Decisional autonomy	My job allows me to decide with a certain degree of autonomy on the planning and scheduling of activities
	2. My job allows me to decide with a certain degree of autonomy on the time to devote to my activities
	3. My job allows me to decide with a certain degree of autonomy on the programming and planning of the activities I carry out
Colleagues’ support	Colleagues give me the help and support I need
	2. At work my colleagues show me the respect I deserve
	3. Colleagues are willing to listen to my work problems
	4. If work becomes difficult, I can count on the help of my colleagues
Support from administrative staff	Please indicate how supported you feel regarding: accounting aspects
	2. Please indicate how supported you feel regarding: teaching management
	3. Please indicate how supported you feel regarding: the management aspects of research projects (e.g., laboratories, etc.)
Hierarchical superiors’ support	I receive supportive information from my Head of Dept. who helps me in the work I do
	2. I can rely on my Head of Dept. if I have any problems at work
	3. If something at work disturbed or bothered me, I can talk to my Head of Dept. about it
Students’ support	Students recognize the effort I put into my work
	2. Students are on the same wavelength as me
	3. Students explicitly appreciate the way I work
Participation	In my department, I have influence on decisions that affect my scientific sector
	2. In my department, I have influence on the decision-making processes
	3. In my department, I have influence on affecting organizational changes
Comfort of teaching environments	1. Assess the level of appropriateness of the following aspects of your working environment: The state of the teaching facilities
	2. Assess the level of appropriateness of the following aspects of your working environment: The state of the teaching equipment
Comfort of research environments	Assess the level of appropriateness of the following aspects of your working environment: The state of the research facilities
	2. Assess the level of appropriateness of the following aspects of your working environment: The state of the research tools and equipment (hardware, software, machinery)
Mediators/moderators	
Working excessively	I seem to be in a hurry and racing against the clock
	2. I stay busy and keep many irons in the fire
	3. I find myself doing two or three things at one time
	4. I find myself continuing to work even when others tell me to stop
	5. I spend more time working than socializing with friends or hobbies or leisure activities
Working compulsively	It’s important for me to work hard even when I don’t like what I’m doing
	2. I feel that there’s something inside me that drives me to work hard
	3. I feel obliged to work hard, even when it’s not enjoyable
	4. I feel guilty when I am not working on something
	5. It is hard for me to relax when I am not working
Outcomes	
Emotional exhaustion	I feel emotionally worn out by my job
	2. I feel exhausted at the end of a workday
	3. I feel tired when I get up in the morning and have to face another day of work
	4. Working all day is really an effort for me
	5. I feel exhausted by my work
Work engagement	In my work I feel full of energy
	2. In my work I feel strong and vigorous
	3. When I get up in the morning, I feel like going to work
	4. I am proud of the work I do
	5. I am enthusiastic about my work
	6. My work inspires me

## References

[1] Kinman G., Wray S., Burke R.J., Pignata S. (2020). Well-being in academic employees—A benchmarking approach. Handbook of Research on Stress and Well-Being in the Public Sector.

[2] Wray S., Kinman G. (2022). The challenges of COVID-19 for the well-being of academic staff. Occup. Med..

[3] Urbina-Garcia A. (2020). What do we know about university academics’ mental health? A systematic literature review. Stress Health.

[4] Franke F. (2015). Is work intensification extra stress?. J. Pers. Psych..

[5] Chuan-Chiew G., Hwa M.A.C., Teh G.M. (2018). Work intensification and turnover intention in academia: The mediating role of work-life balance. J. Asian Sci. Res..

[6] Kinman G., Johnson S. (2019). Special section on well-being in academic employees. Int. J. Stress Manag..

[7] Szromek A.R., Wolniak R. (2020). Job satisfaction and problems among academic staff in higher education. Sustainability.

[8] Daumiller M., Rinas R., Hein J., Janke S., Dickhäuser O., Dresel M. (2021). Shifting from face-to-face to online teaching during COVID-19: The role of university faculty achievement goals for attitudes towards this sudden change, and their relevance for burnout/engagement and student learning. Comp. Hum. Behav..

[9] Salanova M., Lorens S., Cifre E. (2013). The dark side of technologies: Technostress among users of information and communication technologies. Int. J. Psychol..

[10] Li L., Wang X. (2020). Technostress inhibitors and creators and their impacts on university teachers’ work performance in higher education. Cogn. Techn. Work..

[11] Jena R. (2015). Technostress in ICT enabled collaborative learning environment: An empirical study among Indian academicians. Comp. Hum. Behav..

[12] Salo M., Pirkkalainen H., Koskelainen T. (2019). Technostress and social networking services: Explaining users’ concentration, sleep, identity, and social relation problems. Inform. Syst. J..

[13] Bakker A.B., Demerouti E. (2017). Job demands–resources theory: Taking stock and looking forward. J. Occup. Health Psychol..

[14] Bowling N.A., Kirkendall C., Houdmont J., Leka S., Sinclair R.R. (2012). Workload: A review of causes, consequences, and potential interventions. Contemporary Occupational Health Psychology: Global Perspectives on Research and Practice.

[15] Xie J.L., Schaubroeck J., Lam S.S.K. (2008). Theories of job stress and the role of traditional values: A longitudinal study in China. J. Appl. Psychol..

[16] Adil A., Kamal A. (2020). Authentic leadership and psychological capital in job demands-resources model among Pakistani university teachers. Int. J. Leadersh. Educ..

[17] Darabi M., Macaskill A., Reidy L. (2019). A qualitative study of the UK academic role: Positive features, negative aspects and associated stressors in a mainly teaching-focused university. J. Furth. High. Educ..

[18] Innstrand S.T., Christensen M., Undebakke K.G., Svarva K. (2015). The presentation and preliminary validation of KIWEST using a large sample of Norwegian university staff. Scand. J. Public Health.

[19] Rahoo L.A., Raza S.A., Arain M.W., Memon M. (2017). A study on occupational stress among faculty members in private institutes of Hyderabad. Sindh. Res. Hum. Soc. Sci..

[20] Salami S.O. (2011). Job stress and burnout among lecturers: Personality and social support as moderators. Asian Soc. Sci..

[21] Simons A., Munnik E., Frantz J., Smith M. (2019). The profile of occupational stress in a sample of health profession academics at a historically disadvantaged university in South Africa. S. Afr. J. Higher Educ..

[22] Ishaq R., Mahmood A. (2017). Relationship between job stress and employee burnout: The moderating role of self-efficacy for university teachers. J. Res. Reflect. Educ..

[23] García-González M.A., Torrano F., García-González G. (2020). Analysis of Stress Factors for Female Professors at Online Universities. Int. J. Env. Res. Public Health.

[24] Ogbonna E., Harris L.C. (2004). Work intensification and emotional labour among UK university lecturers: An exploratory study. Organ. Stud..

[25] Kinman G. (2001). Pressure Points: A review of research on stressors and strains in UK academics. Educ. Psychol..

[26] Charoensukmongkol P., Phungsoonthorn T. (2020). The Interaction Effect of Crisis Communication and Social Support on The Emotional Exhaustion of University Employees during the COVID-19 Crisis. Int. J. Bus. Commun..

[27] Mudrak J., Zabrodska K., Kveton P., Jelinek M., Blatny M., Solcova I., Machovcova K. (2018). Occupational well-being among university faculty: A job demands-resources model. Res. High. Educ..

[28] Han J., Yin H., Wang J. (2020). Examining the relationships between job characteristics, emotional regulation and university teachers’ well-being: The mediation of emotional regulation. Front. Psychol..

[29] Loera B., Martini M., Viotti S., Converso D. (2016). Users’ support as a social resource in educational services: Construct validity and measurement invariance of the User-Initiated Support Scale (UISS). Front. Psych..

[30] Gray S. (2015). Culture clash or ties that bind? What Australian academics think of professional staff. J. High. Educ. Policy Manag..

[31] Morgeson F.P., Humphrey S.E. (2006). The work design questionnaire (WDQ): Developing and validating a comprehensive measure for assessing job design and the nature of work. J. Appl. Psychol..

[32] Badri S.K.Z., Panatik S.A. (2020). The roles of job autonomy and self-efficacy to improve academics’ work-life balance. Asian Acad. Manag. J..

[33] Bakker A.B., Demerouti E. (2007). The Job Demands-Resources model: State of the art. J. Man. Psychol..

[34] Demerouti E., Bakker A.B., Nachreiner F., Schaufeli W.B. (2001). The Job Demands-Resources model of burnout. J. Appl. Psychol..

[35] Schaufeli W.B. (2017). Applying the Job Demands-Resources model. Organ. Dyn..

[36] Maslach C. (2001). What have we learned about burnout and health?. Psychol. Health.

[37] Demerouti E., Bakker A.B. (2011). The Job Demands-Resources model: Challenges for future research. S. Afr. J. Ind. Psychol..

[38] Schaufeli W.B., Bakker A.B. (2004). Job demands, job resources, and their relationship with burnout and engagement: A multi-sample study. J. Organ. Behav..

[39] Bakker A.B., Demerouti E., Sanz-Vergel A.I. (2014). Burnout and work engagement: The JD-R approach. Annu. Rev. Organ. Psychol. Organ. Behav..

[40] Luthans F., Youssef C.M. (2007). Emerging positive organizational behavior. J. Manag..

[41] Xanthopoulou D., Bakker A.B., Demerouti E., Schaufeli W.B. (2007). The role of personal resources in the job demands-resources model. Int. J. Stress Manag..

[42] Carleton E.L., Barling J., Trivisonno M. (2018). Leaders’ trait mindfulness and transformational leadership: The mediating roles of leaders’ positive affect and leadership self-efficacy. Can. J. Behav. Sci..

[43] Bakker A.B., Demerouti E., Chen P.Y., Cooper C.L. (2014). Job demands: Resources theory. Work and Wellbeing: A Complete Reference Guide.

[44] Lesener T., Gusy B., Wolter C. (2019). The job demands-resources model: A meta-analytic review of longitudinal studies. Work Stress.

[45] Christensen M., Dyrstad J.M., Innstrand S.T. (2018). Academic work engagement, resources and productivity: Empirical evidence with policy implications. Stud. High. Educ..

[46] Converso D., Loera B., Molinengo G., Viotti S., Guidetti G. (2018). Not All Academics Are Alike: First Validation of the Academics’ Quality of Life at Work Scale (AQoLW). Front. Psychol..

[47] Winefield A.H., Gillespie N., Stough C., Dua J., Hapuarachchi J., Boyd C. (2003). Occupational stress in Australian university staff: Results from a national survey. Int. J. Stress Manag..

[48] Curran P.J., West S.G., Finch J.F. (1996). The robustness of test statistics to nonnormality and specification error in confirmatory factor analysis. Psychol. Methods.

[49] Kline R.B. (2016). Principles and Practice of Structural Equation Modeling.

[50] Chen F.F. (2007). Sensitivity of goodness of fit indexes to lack of measurement invariance. Struct. Equ. Modeling Multidiscip. J..

[51] Boyd C.M., Bakker A.B., Pignata S., Winefield A.H., Gillespie N., Stough C. (2011). A longitudinal test of the job demands-resources model among Australian university academics. Appl. Psych..

[52] Edwards J.A., Webster S., Van Laar D., Easton S. (2008). Psychometric analysis of the UK health and safety executive’s management standards work-related stress indicator tool. Work Stress.

[53] Toderi S., Balducci C., Edwards J.A., Sarchielli G., Broccoli M., Mancini G. (2013). Psychometric properties of the UK and Italian versions of the HSE Stress Indicator Tool: A crosscultural investigation. Eur. J. Psychol. Assess..

[54] Melin M., Astvik W., Bernhard-Oettel C. (2014). New work demands in higher education. A study of the relationship between excessive workload, coping strategies and subsequent health among academic staff. Qual. High. Educ..

[55] Ghislieri C., Emanuel F., Molino M., Cortese C.G., Colombo L. (2017). New technologies smart, or harm work-family boundaries management? Gender differences in conflict and enrichment using the JD-R theory. Front. Psych..

[56] De Carlo N.A., Falco A., Capozza D. (2008). Test for the Assessment of Work-Related Stress Risk in the Organizational Well-Being Perspective, Q-Bo.

[57] Dormann C., Zapf D. (2004). Customer-related social stressors and burnout. J. Occup. Health Psychol..

[58] Carlson D.S., Kacmar K.M., Williams L.J. (2000). Construction and initial validation of a multidimensional measure of work–family conflict. J. Voc. Behav..

[59] Colquitt J.A. (2001). On the dimensionality of organizational justice: A construct validation of a measure. J. Appl. Psych..

[60] Spagnoli P., Farnese M.L., D’Olimpio F., Millefiorini A., Kovalchuk L.S. (2017). Psychometric properties of the Italian version of Colquitt’s Organizational Justice Scale (OJS). Int. J. Organ. Anal..

[61] Kristensen T.S., Hannerz H., Høgh A., Borg V. (2005). The Copenhagen Psychosocial Questionnaire-a tool for the assessment and improvement of the psychosocial work environment. Scand. J. Work Environ. Health.

[62] Balducci C., Avanzi L., Consiglio C., Fraccaroli F., Schaufeli W. (2017). A cross-national study on the psychometric quality of the Italian version of the Dutch Work Addiction Scale (DUWAS). Eur. J. Psychol. Assess..

[63] Zimmermann B.K., Dormann C., Dollard M.F. (2011). On the positive aspects of customers: Customer-initiated support and affective crossover in employee–customer dyads. J. Occup. Organ. Psych..

[64] Karasek R. (1985). Job Content Instrument Questionnaire and User’s Guide.

[65] Balducci C., Fraccaroli F., Schaufeli W.B. (2010). Psychometric properties of the Italian version of the Utrecht Work Engagement Scale (UWES-9). Eur. J. Psych. Assess..

[66] Kinman G., Jones F. (2008). Effort-reward imbalance, over-commitment and work-life conflict: Testing an expanded model. J. Man. Psychol..

[67] Pignata S., Biron C., Dollard M.F., Peeters M., de Jonge J., Taris T.W. (2014). Managing psychosocial risks in the workplace: Prevention and intervention. People at Work: An Introduction to Contemporary Work Psychology.

[68] Potter R.E., Zadow A., Dollard M., Pignata S., Lushington K. (2021). Digital communication and health wellbeing in universities: A double-edged sword. J. Higher Educ. Policy Manag..

[69] Potter R.E., Dollard M., Pignata S., Zadow A., Lushington K. (2022). Review of practice policy strategies for managing digital communication and ICT use in Australian universities. Comp. Human Behav..

[70] Dimitrov R.S. (2010). Inside Copenhagen: The state of climate governance. Glob. Environ. Politics.

[71] Chien C.C., Yao G., Michalos A.C. (2014). Norms. Encyclopedia of Quality of Life and Well-Being Research.

[72] White M.P., Alcock I., Grellier J., Wheeler B.W., Hartig T., Warber S.L., Bone A., Depledge M.H., Fleming L.E. (2019). Spending at least 120 minutes a week in nature is associated with good health and wellbeing. Sci. Rep..

